# Dynamic Incidence Angle Effects of Non-Spherical Raindrops on Rain Attenuation and Scattering for Millimeter-Wave Fuzes

**DOI:** 10.3390/s25216779

**Published:** 2025-11-05

**Authors:** Bing Yang, Kaiwei Wu, Yanbin Liang, Shijun Hao, Zhonghua Huang

**Affiliations:** School of Mechatronical Engineering, Beijing Institute of Technology, Beijing 100081, China; 3120215164@bit.edu.cn (B.Y.); 3120205163@bit.edu.cn (K.W.); 3120195127@bit.edu.cn (Y.L.); 3120215165@bit.edu.cn (S.H.)

**Keywords:** millimeter-wave fuzes, non-spherical raindrop, IIM T-matrix method, incidence angle, attenuation coefficient

## Abstract

The dynamic variation of the incidence angle between the millimeter-wave (MMW) fuzes and non-spherical raindrops significantly affects detection performance. To address this issue, the influence of incidence angle on attenuation coefficient, volume reflectivity, and the signal-to-clutter-plus-noise ratio (SCNR) is systematically analyzed by employing the realistic raindrop morphology described by the Beard and Chuang (BC) model and the invariant imbedding (IIM) T-matrix method. By integrating worst-case analysis, the critical incidence angle corresponding to the most severe performance degradation is identified, and the corresponding attenuation coefficient, volume reflectivity, and SCNR values are reconstructed. Numerical simulations demonstrate that for the BC model, the most severe impact on MMW signal propagation occurs at an incidence angle of 180°. Under this condition, the reconstructed attenuation coefficient and volume reflectivity increase by 45.88% and 28.27%, respectively, while the SCNR decreases by 27.35% at 60 GHz operating frequency and 100 mm/h rainfall rate, compared to the spherical raindrop model. This study provides a theoretical basis for calibrating design margins and optimizing anti-interference strategies for MMW fuzes operating in complex meteorological conditions.

## 1. Introduction

The millimeter-wave (MMW) fuze is a detection system that employs MMW signals for precise target identification [1]. Owing to its high resolution and robust anti-electromagnetic interference capabilities, MMW fuzes have become a cornerstone of modern precise distance measurement systems [2]. Under rainfall conditions, raindrop-induced attenuation significantly diminishes the echo power of MMW signals. Simultaneously, backscattering from raindrops elevates the rain clutter power within the fuze’s echo return, thereby imposing substantial operational constraints on MMW fuze performance [3,4]. It is imperative to conduct a systematic investigation into the attenuation and backscattering effects imposed by raindrops on MMW fuze systems.

Raindrop shape and its spatial orientation are critical factors that collectively govern the attenuation and scattering behavior of MMW signals [5]. Extensive research has been conducted to characterize raindrop shapes. Pioneering work by Ryde [6] in the 1940s employed spherical raindrops to calculate electromagnetic cross-sections, while Spilhaus [7] proposed an ellipsoidal approximation for raindrop shape. Subsequent experimental advancements by Pruppacher [8], utilizing high-speed cameras in wind tunnel studies, led to the development of the Pruppacher-Pitter model. Building upon this foundation, Beard and Chuang (BC) [9] incorporated the stress equilibrium among surface tension, hydrostatic pressure, and aerodynamic pressure to establish the more physically representative BC model, which characterizes raindrops as oblate spheroids with a flattened base. Recent validation studies by Feng Wanyue [10] and Merhala Thurai [11], employing linear array charge-coupled device scanning detection and two-dimensional video disdrometer techniques, respectively, have corroborated that the BC model provides a superior representation of actual raindrop shapes. Substantial evidence confirms the non-spherical morphology of raindrops, underscoring the necessity to investigate their attenuation and scattering characteristics utilizing the realistic BC model.

Furthermore, the spatial orientation effect of raindrops is governed by the range and distribution of their inclination angles, which has attracted considerable research attention. The raindrop inclination angle is defined as the angle between the rotational axis of the raindrop and the vertical direction. Under ideal conditions, raindrops maintain a vertical orientation; however, natural rainfall is subject to disturbances such as wind and turbulence, leading to deviations in their orientation [12]. Studies by Kenneth V. Beard [13] have demonstrated that the distribution of raindrop inclination angles follows a Gaussian pattern. Subsequent investigations by Hendry [5] and Huang [14], utilizing linear polarization radar and differential reflectivity measurements, further confirmed that the mean inclination angle is approximately 0°, with a standard deviation ranging from 4° to 15°. Empirical observations by Hu Yuntao [15] based on field measurements indicate that the inclination angle can vary within a broader range of [−45°, 45°] under realistic atmospheric conditions. Findings reveal that raindrops do not always align with a vertical orientation, and their inclination angles follow a non-uniform distribution.

Under the conditions of irregular raindrop shapes and non-uniform distribution of raindrop inclination angles, the incidence angle of MMW fuzes on raindrops becomes a critical yet underexplored variable governing the fuze’s echo characteristics. The incidence angle is primarily determined by the raindrop inclination angle, the axial direction of the fuze’s detection beam, and the beam width. During flight, the orientation of the fuze undergoes continuous variation. Research by Peng Qimeng [16] indicates that the angle between the fuze’s detection direction and the vertical axis may range from 45° to 150°. Furthermore, to accommodate wider impact angles, fuze systems typically employ wide beam configurations. Studies by Lei Jun [17] and Xin Liao [18] report that the 3 dB beamwidth of a MMW fuze can reach up to 120°. Consequently, the incidence angle of raindrops relative to the MMW fuze can span an extensive range during the flight trajectory.

The large-scale variation in the raindrop incidence angle induces strong temporal fluctuations and chaotic dynamics in both backscattering and attenuation characteristics. This phenomenon poses a fundamental challenge to signal processing in such systems. Consequently, investigating the influence of non-spherical raindrop incidence angles on the attenuation and scattering effects in MMW fuzes is of critical importance. Furthermore, to ensure the reliability and stability of MMW fuzes throughout the detection process, it is critical to analyze scattering and attenuation properties under extreme conditions. Accordingly, this study adopts a worst-case analysis approach [19] to identify the incidence angle that leads to the most severe performance degradation. The attenuation and scattering parameters of the MMW fuzes are subsequently recalculated under this critical angle to evaluate its operational limits.

Additionally, the numerical methods employed for solving the electromagnetic scattering properties of raindrops play a critical role in determining the accuracy of computational results. Commonly used techniques include classical Mie theory [20], the point-matching method [21], the conventional T-matrix method [22], and the discrete dipole approximation (DDA) [23]. Mie theory is strictly applicable to ideal spherical particles; the point-matching method handles regularly shaped particles; the conventional T-matrix method can model rotationally symmetric non-spherical particles but suffers from numerical instability at large size parameters, while DDA accommodates arbitrarily complex shapes at the expense of computational efficiency. To address these limitations, the IIM T-matrix method has garnered significant attention [24,25,26]. This method is founded on a volume integral formulation for solving the T-matrix, which eliminates the dependency on boundary conditions that constrain conventional T-matrix solutions. Owing to this fundamental characteristic, the IIM T-matrix method can enable accurate and efficient computations for particles of arbitrary shape and orientation. The computational accuracy and reliability of the IIM T-matrix method have been validated through Li Hai’s [25] comparison with the conventional T-matrix algorithm for spheroidal particles and Hu Shuai’s [27] comparative analysis with the DDA for irregularly shaped particles. Capitalizing on these advantages, the present study employs the IIM T-matrix method as the core numerical tool to systematically investigate the attenuation and scattering properties of non-spherical raindrops under various incidence angles.

Therefore, to investigate the impact of dynamic incidence angles of non-spherical raindrops on MMW fuzes in rainfall environments, this study systematically computed the attenuation and scattering properties of three typical raindrop morphologies—spherical model (SP model), approximate ellipsoidal model (AE model), and the BC model—across a range of incidence angles using the IIM T-matrix method. A quantitative analysis was conducted to evaluate the fluctuations in the attenuation coefficient, volume reflectivity, and the signal-to-clutter-plus-noise ratio (SCNR) of the fuzes echo under different incidence angles. Furthermore, based on a worst-case analysis methodology, the specific incidence angle leading to the most severe performance degradation was identified. At this critical raindrop incidence angle, the attenuation coefficient, volume reflectivity, and SCNR of the MMW fuzes were recalculated. This integrated approach, combining a realistic raindrop model with a worst-case analysis under dynamic incidence angles, provides a novel and more reliable framework for assessing MMW fuze performance beyond conventional static or spherical-model-based analyses.

The remainder of this paper is organized as follows: Section 2 presents the theoretical framework and methodology. In Section 3, the attenuation characteristics, backscattering properties, and SCNR of the BC model under different incidence angles are investigated. The specific incidence angle is determined that leads to the most severe degradation in MMW fuzes performance. Building on the critical incidence angle, Section 4 recalculates the attenuation coefficient, volume reflectivity, and SCNR. A comparative analysis among the BC model, SP model, and AE model is also provided. Finally, Section 5 concludes the paper.

## 2. Theory and Methods

### 2.1. Physical Characteristics of Raindrop Particles

**Figure 1:**
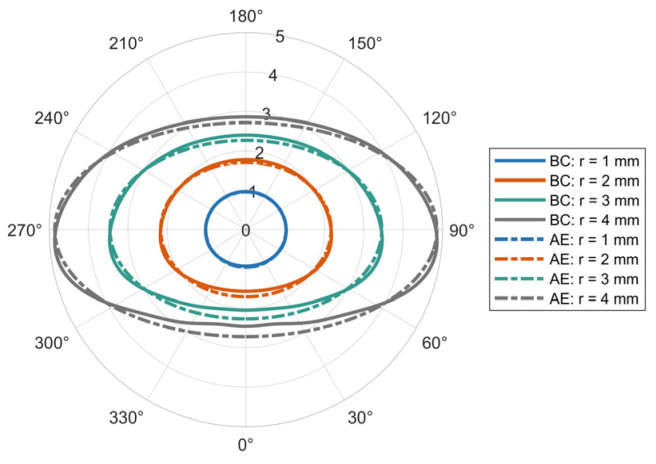
Raindrop particle shape: BC model and AE model.

The natural shape of falling raindrops approximates an oblate spheroidal geometry, characterized by a rounded upper portion and a flattened base. This morphology is accurately represented by the BC model, which is given below:

(1) $$r\left(\varphi \right)=r_{0}\left[1+\sum_{n=1}^{10}c_{n}\cos\left(n\varphi \right)\right],$$

where $r_{0}$ denotes the equivalent radius of the raindrop and φ is the polar angle. The coefficients $c_{n}$ correspond to the shape parameters, with their specific numerical values provided in [9]. In earlier research, raindrop shapes were commonly approximated as AE model or SP model to streamline computational procedures. For comparative purposes, the AE model is provided as follows [28]:

(2) $$\frac{b}{a}=0.9971+0.2193D-3.5105D^{2}+5.0746D^{3}-2.3559D^{4},$$

where b denotes the semi-axis of rotation in the AE model, while a represents the semi-axis along the horizontal direction, D is the equivalent diameter. A schematic comparison between the BC model and AE model is provided in Figure 1.

Figure 1 indicates that for small raindrop radius, both the BC and AE models approximate a spherical geometry. As the radius increases, the BC model progressively develops a flattened base morphology.

The drop size distribution (DSD) characterizes the number concentration of raindrops per unit volume as a function of their equivalent spherical diameter. Among the various models proposed, the Marshall-Palmer distribution has been extensively adopted in meteorological and electromagnetic wave propagation studies. The Marshall-Palmer distribution is expressed as follows:

(3) $$N\left(I,D\right)=8000\exp\;\left(-4.1I^{-0.21}D\right),$$

where $N\left(I,D\right)$ denotes the number concentration of raindrops per unit size interval, and I represents the rainfall intensity in millimeters per hour.

The refractive index of raindrops characterizes the optical properties of MMW signals propagating through precipitation media. As a function of both temperature and frequency, the refractive index in this study is calculated utilizing the Ray’s model [29], expressed as follows:

(4) $$\left\{\begin{array}{l} \varepsilon^{\prime }=\varepsilon_{\infty }+\frac{\left(\varepsilon_{s}-\varepsilon_{\infty }\right){\left(1+\frac{\lambda_{s}}{\lambda }\right)}^{1-\mu }\sin\left(\mu \frac{\pi }{2}\right)}{1+{2\left(\frac{\lambda_{s}}{\lambda }\right)}^{1-\mu }\sin\left(\mu \frac{\pi }{2}\right)+{\left(\frac{\lambda_{s}}{\lambda }\right)}^{2\left(1-\mu \right)}}\\ \varepsilon^{\prime \prime }=\frac{\sigma \lambda }{18.8496\times 10^{10}}+\frac{\left(\varepsilon_{s}-\varepsilon_{\infty }\right){\left(\frac{\lambda_{s}}{\lambda }\right)}^{1-\mu }\cos\left(\mu \frac{\pi }{2}\right)}{1+{2\left(\frac{\lambda_{s}}{\lambda }\right)}^{1-\mu }\sin\left(\mu \frac{\pi }{2}\right)+{\left(\frac{\lambda_{s}}{\lambda }\right)}^{2\left(1-\mu \right)}} \end{array}\right.,$$

where λ denotes the wavelength of the MMW signal, which is determined by the propagation velocity of the electromagnetic wave c and the operating frequency of the MMW fuzes f, according to the relation λ=c/f. The values of additional parameters are specified as follows:

(5) $$\left\{\begin{array}{l} \varepsilon_{s}=78.54\left[\begin{array}{c} 1-4.579\times 10^{-3}\left(T-25\right)+1.19\times 10^{-5}{\left(T-25\right)}^{2}\\ -2.8\times 10^{-8}{\left(T-25\right)}^{3} \end{array}\right]\\ \varepsilon_{\infty }=5.27134+2.16474\times 10^{-2}\;T-1.31198\times 10^{-3}\;T^{2}\\ \begin{array}{l} \mu =-\frac{16.8129}{T+273}+6.09265\times 10^{-2}\\ \lambda_{s}=3.3836\times 10^{-4}\exp\left(\frac{2513.98}{T+273}\right)\\ \sigma =12.5664\times 10^{-4} \end{array} \end{array}\right.,$$

where T represents the ambient temperature, with units of degrees Celsius (°C).

### 2.2. Scale of Raindrop Incidence Angles in MMW Fuzes

Owing to the non-uniform orientation of raindrop particles, the attenuation and scattering of MMW signals exhibit significant dependence on the incidence angle. Additionally, the flight trajectory of a MMW fuze—comprising ascending, level-flight, and descending phases—progressively alters the direction of signal transmission. This subsection consequently focuses on determining the operational scale of incidence angles between the fuzes and raindrops. A schematic diagram illustrating the fuzes flight path relative to the raindrop coordinate system is presented below.

**Figure 2:**
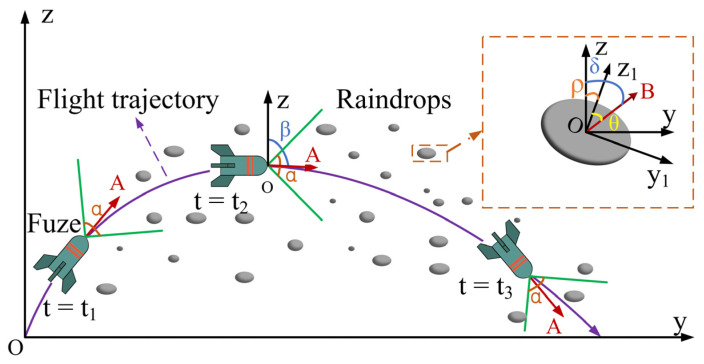
Schematic of the MMW fuze’s flight trajectory and raindrop incidence angles.

In Figure 2, the flight trajectory of the MMW fuze is represented as the purple curve. Time instances $t_{1}$, $t_{2}$, and $t_{3}$ correspond to the ascending, level-flight, and descending phases of the trajectory, respectively. The parameter α denotes the 3 dB vertical beamwidth of the MMW fuze. To simplify the computational model, the horizontal and vertical 3 dB beamwidths are assumed to be equal. The angle between the fuze’s forward direction $\vec{OA}$ and the z-axis is defined as β, with the z-axis oriented normal to the ground plane and upward throughout. The coordinate system for the raindrop particle is illustrated in the upper-right corner. Within this system, the symmetry axis of the raindrop particle is designated as $z_{1}$, and the horizontal axis is denoted as $y_{1}$. The angle ρ between the raindrop’s symmetry axis $z_{1}$ and the z-axis represents the inclination angle of the raindrop. The incident direction of the MMW signal relative to the raindrop is indicated by the red solid line $\vec{OB}$. The angle between this incident direction and the z-axis is defined as δ, while the angle with the $z_{1}$ axis is defined as θ. The parameter θ adenotes the incidence angle of the MMW signal relative to the raindrop, defined as the angle with respect to the raindrop’s rotation axis $z_{1}$. This definition facilitates the subsequent calculation of raindrop attenuation and scattering properties across various incidence angles.

Owing to the rotational symmetry of raindrop particles, the analysis of the incidence angle can be confined to the yoz plane without loss of generality. The operational range of this incidence angle is determined by synthesizing the fuze’s flight trajectory with the vertical 3 dB beamwidth of its antenna and the raindrop inclination angle, expressed as follows:

(6) $$\theta =\delta -\rho ,\delta \in \left[\beta -\frac{\alpha }{2},\beta +\frac{\alpha }{2}\right],$$

The angle between the fuze and the Z-axis is assumed to be 45° at launch and 150° during descent [16], which corresponds to the parameter β ranging from 45° to 150°. The vertical 3 dB beamwidth α is set to 120°. The inclination angle ρ of raindrops varies within the range of −45° to 45° [15]. Consequently, the incidence angle θ of the MMW signal relative to raindrops spans from 0° to 180°.

### 2.3. IIM T-Matrix Method for Computing the Raindrop Attenuation and Backscattering Characteristics

**Figure 3:**
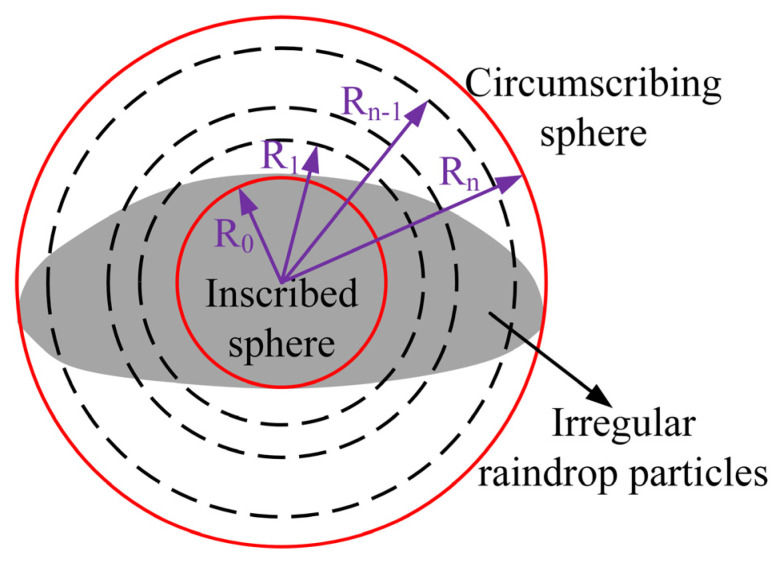
Flowchart of the IIM T-matrix Computational Algorithm.

The IIM T-matrix method represents an improved variant of the classical T-matrix framework, incorporating modifications to the treatment of boundary conditions. This adaptation effectively mitigates the elevated numerical errors associated with modeling large-sized particles, leading to its increasing adoption in recent applications. The computational procedure of the IIM T-matrix method initiates with the application of Mie scattering theory to derive the electromagnetic response of the spherical region inscribed within an irregularly shaped particle. Subsequently, a stratified iterative algorithm is employed to compute the T-matrix for the volumetric domain bounded by the inscribed and circumscribed spheres. The overall T-matrix of the irregular particle, synthesized from these steps, is then utilized for the analysis of its attenuation and scattering characteristics. A schematic illustration delineating this computational workflow is presented in Figure 3.

The iterative formulation employed for computing the IIM T-matrix method is expressed as follows:

(7) $$\overset{̿}{T}(r_{n})={\overset{̿}{Q}}_{11}(r_{n})+(\overset{̿}{I}+{\overset{̿}{Q}}_{12}(r_{n}))[\overset{̿}{I}-\overset{̿}{T}(r_{n-1}){\overset{̿}{Q}}_{22}(r_{n}){]}^{-1}\overset{̿}{T}(r_{n-1})[\overset{̿}{I}+{\overset{̿}{Q}}_{21}(r_{n})],$$

where $\overset{̿}{T}(r_{n})$ and $\overset{̿}{T}(r_{n-1})$ denote the T-matrices corresponding to the *n*th and (*n* − 1)th concentric spherical layers, respectively. $\overset{̿}{I}$ represents the identity matrix. The matrices ${\overset{̿}{Q}}_{11}\left(r_{n}\right)$, ${\overset{̿}{Q}}_{12}\left(r_{n}\right)$, ${\overset{̿}{Q}}_{21}\left(r_{n}\right)$, and ${\overset{̿}{Q}}_{22}\left(r_{n}\right)$ are derived from the Q-matrices $\overset{̿}{Q}\left(r_{n}\right)$, Bessel matrices $\overset{̿}{J}\left(r_{n}\right)$ and Hankel matrices $\overset{̿}{H}\left(r_{n}\right)$, given as

(8) $$\left\{\begin{array}{c} {\overset{̿}{Q}}_{11}\left(r_{n}\right)=ik{\overset{̿}{J}}^{T}\left(r_{n}\right)\overset{̿}{Q}\left(r_{n}\right)\overset{̿}{J}\left(r_{n}\right)\\ {\overset{̿}{Q}}_{12}\left(r_{n}\right)=ik{\overset{̿}{J}}^{T}\left(r_{n}\right)\overset{̿}{Q}\left(r_{n}\right)\overset{̿}{H}\left(r_{n}\right)\\ \begin{array}{c} {\overset{̿}{Q}}_{21}\left(r_{n}\right)=ik{\overset{̿}{H}}^{T}\left(r_{n}\right)\overset{̿}{Q}\left(r_{n}\right)\overset{̿}{J}\left(r_{n}\right)\\ {\overset{̿}{Q}}_{22}\left(r_{n}\right)=ik{\overset{̿}{H}}^{T}\left(r_{n}\right)\overset{̿}{Q}\left(r_{n}\right)\overset{̿}{H}\left(r_{n}\right) \end{array} \end{array}\right.,$$

where the Q-matrices $\overset{̿}{Q}\left(r_{n}\right)$ are calculated by

(9) $$\overset{̿}{Q}\left(r_{n}\right)=\omega_{n}{\left[\overset{̿}{I}-\omega_{n}\overset{̿}{U}\left(r_{n}\right)\overset{̿}{g}\left(r_{n},r_{n}\right)\right]}^{-1}\overset{̿}{U}\left(r_{n}\right),$$

where $\omega_{n}$ represents the weight factor in the Gauss-Legendre quadrature formula. The Matrice $\overset{̿}{g}\left(r_{n},r_{n}\right)$, Bessel matrices $\overset{̿}{J}\left(r_{n}\right)$ and Hankel matrices $\overset{̿}{H}\left(r_{n}\right)$ are superdiagonal matrices, with their respective diagonal elements defined by the submatrices ${\overset{̿}{g}}_{n}(r_{n},r_{n})$, ${\overset{̿}{J}}_{n}(kr_{n})$, and ${\overset{̿}{J}}_{n}(kr_{n})$, as given by the following expressions:

(10) $${\overset{̿}{J}}_{n}(kr_{n})=\left|\begin{array}{cc} J_{n}(kr_{n}) & 0\\ 0 & \frac{1}{kr_{n}}\frac{d}{d(kr_{n})}(kr_{n}J_{n}(kr_{n}))\\ 0 & \frac{\sqrt{n(n+1)}}{kr_{n}}J_{n}(kr_{n}) \end{array}\right|,$$

(11) $${\overset{̿}{H}}_{n}(kr_{n})=\left|\begin{array}{cc} H_{n}^{\left(1\right)}(kr_{n}) & 0\\ 0 & \frac{1}{kr_{n}}\frac{d}{d(kr_{n})}(H_{n}^{\left(1\right)}(kr_{n}))\\ 0 & \frac{\sqrt{n(n+1)}}{kr_{n}}H_{n}^{\left(1\right)}(kr_{n}) \end{array}\right|,$$

(12) $$\;{\overset{̿}{g}}_{n}(r_{n},r_{n})=\frac{ik({\overset{̿}{J}}_{n}(kr_{n}){\overset{̿}{H}}_{n}(kr_{n})+{\overset{̿}{H}}_{n}(kr_{n}){\overset{̿}{J}}_{n}(kr_{n}))}{2},$$

where $J_{n}(kr_{n})$ denotes the Bessel function of the first kind of order *n*, $H_{n}^{\left(1\right)}(kr_{n})$ is the Hankel function of the first kind of order *n*, $r_{n}$ is the radius of the *n*th spherical layer, and k is wavenumber.

The attenuation and backscattering characteristics induced by raindrops constitute critical factors affecting the detection performance of MMW fuzes. Based on the T-matrix formulation for raindrop particles, the attenuation cross-section $\sigma_{t}$ and the backscattering cross-section $\sigma_{b}$ [25,30] can be derived as:

(13) $$\left\{\begin{array}{l} \sigma_{t,ij}(\theta )=\frac{4\pi }{k}Imag[S_{ij}\left(\vec{\theta },\vec{\theta }\right)]\\ \sigma_{b,ij}(\theta )=4\pi {\left|S_{ij}(-\vec{\theta },\vec{\theta })\right|}^{2} \end{array}\right.,$$

where $S_{ij}(\vec{{Κ}_{1}},\vec{{Κ}_{2}})$ denotes the scattering amplitude matrix, where the vector $\vec{{Κ}_{1}}$ corresponds to the direction of the scattered wave and $\vec{{Κ}_{2}}$ to the direction of the incident wave. The subscripts i and j represent the polarization states of the scattered and incident waves, respectively, with permissible values corresponding to vertical polarization (V) or horizontal polarization (H). Given the predetermined T-matrix, the scattering amplitude matrix can be computed according to Equations (5.11)–(5.17) provided in [31].

The extinction efficiency $Q_{t}(\theta )$ and backscattering efficiency $Q_{b}(\theta )$ of the raindrop are given as:

(14) $$\left\{\begin{array}{c} {Q_{b}(\theta )=\sigma }_{b}(\theta )/(\pi {r_{0}}^{2})\\ {Q_{t}(\theta )=\sigma }_{t}(\theta )/(\pi {r_{0}}^{2}) \end{array}\right.,$$

Furthermore, the attenuation coefficient $\gamma_{t}(\theta )$ and volume reflectivity $\eta_{b}(\theta )$ are given as:

(15) $$\left\{\begin{array}{c} \gamma_{t}(\theta )=4.343\times 10^{-3}\int_{D_{min}}^{D_{max}}\sigma_{t}(\theta )N\left(I,D\right)dD(dB/Km)\\ \eta_{b}(\theta )=\int_{D_{min}}^{D_{max}}\sigma_{b}\left(\theta \right)N\left(I,D\right)dD(m^{2}\cdot m^{-3}) \end{array}\right.,$$

where $D_{max}$ and $D_{min}$ denote the maximum and minimum diameters of the raindrops, respectively.

### 2.4. SCNR of MMW Fuzes in Rainfall Environments

To quantitatively assess the impact of irregular raindrop incidence angles on MMW fuzes, the SCNR has been redefined to incorporate both the attenuation coefficient and the volume reflectivity, thereby providing a more comprehensive performance metric. Under rainfall conditions, assuming a target with a radar cross-section (RCS) of $\sigma_{f}$ for the MMW fuze, the received echo signal power can be expressed as follows [32]:

(16) $$P_{r}(R)=\frac{P_{t}G^{2}\lambda^{2}\sigma_{f}}{{\left(4\pi \right)}^{3}R^{4}}10^{-0.1[\gamma_{t}\left(\theta \right)+\gamma_{t}\left(180-\theta \right)]R},$$

where $P_{r}(R)$ denotes the received echo signal power from the target at distance R, $P_{t}$ represents the transmitted signal power. G indicates the antenna gain, which is assumed to be identical for both transmission and reception.

Correspondingly, the echo signal power of rain clutter at distance R is formulated as follows:

(17) $$P_{c}(R)=\frac{P_{t}G^{2}\lambda^{2}\alpha^{2}∆R\eta }{{256\pi }^{2}R^{2}}10^{-0.1([\left(\theta \right)+\gamma_{t}\left(180-\theta \right)]R},$$

where ∆R denotes the range resolution of the MMW fuzes, which is determined by the speed of electromagnetic wave propagation c and the system bandwidth B according to the relation ∆R=c/(4B). As indicated by this formulation, under constant fuzes system parameters and rainfall conditions, the echo power from rain clutter exhibits an inverse proportionality to the square of the distance. Therefore, rain clutter manifests as a non-homogeneous noise source in the detection process.

The MMW fuze system noise is represented as follows:

(18) $$P_{n}=k_{0}TB_{n}F_{n}L_{s},$$

where $k_{0}$ is the Boltzmann constant, $B_{n}$ denotes the equivalent noise bandwidth, $F_{n}$ represents the noise figure, and $L_{s}$ corresponds to the system loss.

The Constant False Alarm Rate (CFAR) detection scheme is widely employed in fuzes systems for target identification under uniform noise conditions. However, in rainfall environments, the non-uniform spatial distribution of rain-induced clutter leads to high-intensity values in the range cells ahead of the target, which adversely affects target detection and identification performance. Consequently, the SCNR within a single range cell under rainy conditions does not adequately reflect the actual detection difficulty for MMW fuzes. To address this limitation, the SCNR formulation under rainfall conditions has been redefined to incorporate the rain clutter contributions from a specific number of range cells preceding the target cell. In accordance with the number of CFAR reference cells specified in [33], the quantity of rain clutter cells prior to the target range cell is set to 4. The reformulated SCNR is expressed as follows:

(19) $$SINR=\frac{P_{r}(R)}{\sum_{n=0}^{4}P_{c}(R-n*∆R)+P_{n}},$$

## 3. Effects of Incidence Angle Variations in Non-Spherical Raindrops on MMW Fuze Detection

To evaluate the impact of variations in the raindrop incidence angle on MMW fuzes, an investigation was conducted to examine the changes in the attenuation coefficient, volume reflectivity, and SCNR over the incidence angle range of 0° to 180°. The specific parameter values employed in the simulations are summarized in Table 1.

### 3.1. Effects of Incidence Angle Variations in Non-Spherical Raindrops on Attenuation Characteristics

**Figure 4:**
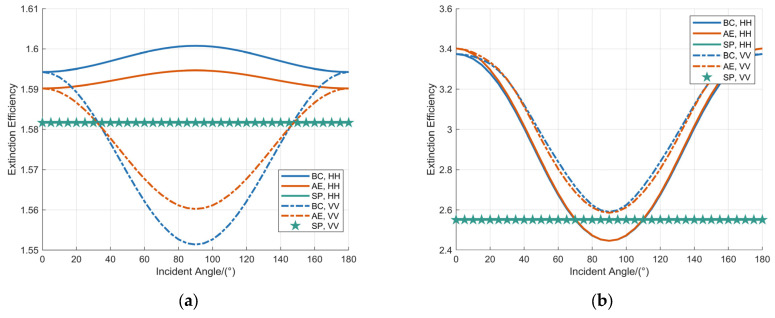
Analysis of the extinction efficiency as a function of the incidence angle for raindrop diameters of: (**a**) 1 mm; (**b**) 8 mm.

The influence of the incidence angle on the attenuation characteristics of MMW signals was investigated. Numerical simulations were performed to analyze the extinction efficiency of raindrops with diameters of 1 mm and 8 mm over the incidence angle range of 0° to 180°. A comparative evaluation of three distinct raindrop shape models—the SP model, the AE model, and the BC model—was conducted. The simulation results are summarized in Figure 4.

Figure 4 illustrates the variation in extinction efficiency as a function of the incidence angle for the SP model, AE model, and BC model. The SP model maintains a constant extinction efficiency across all incidence angles. For raindrops with 8 mm diameter, the extinction efficiencies of both the AE and BC models under various polarization states initially decrease, reach a minimum, and subsequently increase as the incidence angle varies, exhibiting symmetry about 90°. In the case of 1 mm diameter raindrops under horizontal polarization, the extinction efficiencies of the AE and BC models also demonstrate symmetry about 90° but exhibit opposing trends. A comparative analysis reveals that larger raindrops (8 mm) possess a significantly higher extinction efficiency and experience considerably greater fluctuation in response to changes in incidence angle compared to smaller raindrops (1 mm).

**Figure 5:**
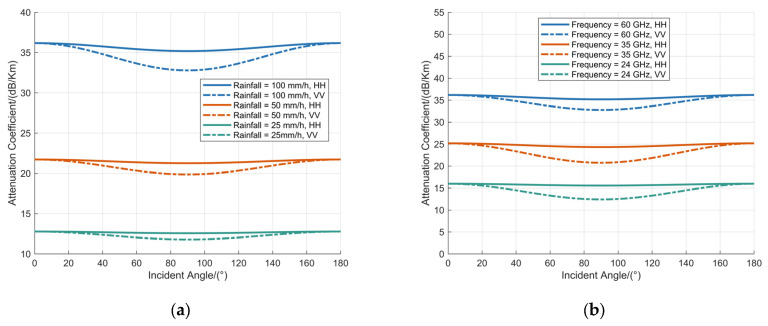
Analysis of attenuation coefficient at various raindrop incidence angles: (**a**) the operating frequency is fixed at 60 GHz; (**b**) the rainfall is fixed at 100 mm/h.

The attenuation coefficient of the raindrop ensemble was simulated. The simulation assumed that during a single detection cycle of the MMW fuze, the incidence angle remained constant for all raindrops within the detection range. Numerical analyses were conducted under two distinct configurations: first, with the fuze operating frequency fixed at 60 GHz, the attenuation coefficient was evaluated for rainfall rates of 100 mm/h, 50 mm/h, and 25 mm/h, as illustrated in Figure 5a; second, with the rainfall rate fixed at 100 mm/h, the attenuation coefficient was simulated for fuze operating frequencies of 60 GHz, 35 GHz, and 24 GHz, as shown in Figure 5b.

Figure 5 depicts the dependence of the raindrop attenuation coefficient on the incidence angle under constant operating frequency and rainfall rate conditions. The attenuation coefficient exhibits a non-monotonic trend, characterized by an initial decrease to a minimum value followed by a subsequent increase as the incidence angle varies. The magnitude of this reduction is less pronounced under horizontal polarization compared to the significant decrease observed under vertical polarization. Furthermore, the attenuation coefficient demonstrates symmetry across the incidence angle range from 0° to 180°.

The attenuation of MMW signals by raindrops is predominantly governed by the magnitude of the attenuation cross-section. When an MMW signal interacts with a raindrop at a specific incidence angle, the attenuation cross-section can be conceptualized as the projected area of the raindrop illuminated by the wave along the propagation direction. Due to the rotational symmetry inherent in the raindrop morphology, as described by the BC model, the attenuation cross-section exhibits symmetric characteristics across the incidence angle range of 0° to 180°. Moreover, the raindrop shape, which approximates an oblate spheroid with a flattened base and a concave structure, results in the projected area—and consequently the attenuation cross-section—reaching its maximum at incidence angles of 0° and 180°. As a result, the attenuation coefficient demonstrates symmetric behavior and attains peak values at these angles, which is consistent with simulation results.

The attenuation coefficient of raindrops exhibits significant dependence on both the rainfall rate and the operating frequency. With the operating frequency fixed at 60 GHz, the maximum attenuation coefficients observed for rainfall rates of 100 mm/h, 50 mm/h, and 25 mm/h are 36.19 dB/km, 21.73 dB/km, and 12.78 dB/km, respectively, across the evaluated range of incidence angles. Additionally, under a constant rainfall rate of 100 mm/h, the maximum attenuation coefficients corresponding to operating frequencies of 60 GHz, 35 GHz, and 24 GHz are 36.19 dB/km, 25.20 dB/km, and 15.99 dB/km, respectively.

### 3.2. Effects of Incidence Angle Variations in Non-Spherical Raindrops on Backscattering Characteristics

**Figure 6:**
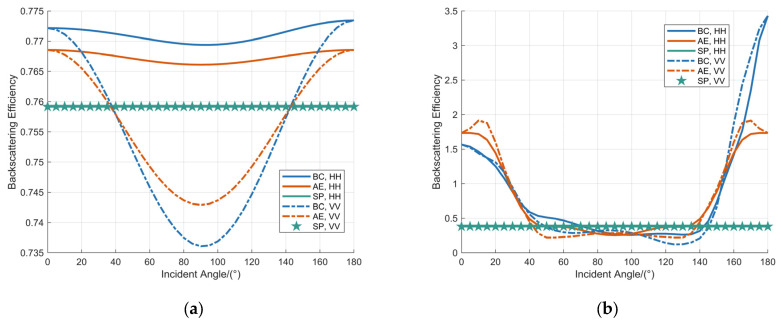
Analysis of the backscattering efficiency as a function of the incidence angle for raindrop diameters of: (**a**) 1 mm; (**b**) 8 mm.

The influence of the incidence angle on the backscattering characteristics of millime-ter-wave signals was investigated. The backscattering efficiency of raindrops with diameters of 1 mm and 8 mm was simulated across various incidence angles. A comparative evaluation of three distinct raindrop shape models—the SP model, the AE model, and the BC model—was conducted. The simulation results are summarized in Figure 6.

Figure 6 demonstrates that for smaller raindrop sizes, the BC model closely approximates an ellipsoidal geometry, resulting in analogous trends in backscattering efficiency variation between the BC model and AE model as the incidence angle changes. Specifically, the backscattering efficiency exhibits a non-monotonic trend, characterized by an initial decrease to a minimum value followed by a subsequent increase across the angular range, demonstrating symmetric behavior about 90°. In contrast, for larger raindrops, the BC model displays heightened shape irregularity. The backscattering efficiency at an incidence angle of 180° is substantially elevated compared to that at 0°. This disparity is attributed to the distinct geometric configurations of the BC model: at 0° incidence, it exhibits a quasi-planar structure, whereas at 180°, it assumes a convex, peaked morphology, leading to an enlarged backscattering efficiency. Remarkably, for large raindrops at 180° incidence, the backscattering efficiencies of the BC model and AE model exceed that of the SP model by factors of 9.03 and 4.55, respectively.

**Figure 7:**
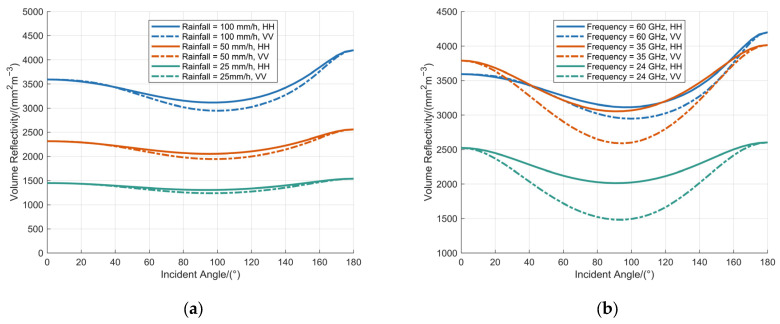
Analysis of volume reflectivity at various raindrop incidence angles: (**a**) the operating frequency is fixed at 60 GHz; (**b**) the rainfall is fixed at 100 mm/h.

The volume reflectivity of the raindrop ensemble was simulated as a function of the incidence angle under two distinct configurations. First, with the operating frequency fixed at 60 GHz, the volume reflectivity was analyzed as a function of incidence angle for rainfall rates of 100 mm/h, 50 mm/h, and 25 mm/h, as presented in Figure 7a. Second, under a constant rainfall rate of 100 mm/h, the volume reflectivity was evaluated for operating frequencies of 60 GHz, 35 GHz, and 24 GHz, as presented in Figure 7b.

Figure 7 demonstrates the variation in volume reflectivity of raindrops, as represented by the BC model, with respect to the incidence angle. The volume reflectivity exhibits a non-monotonic trend, characterized by an initial decrease followed by a subsequent increase as the incidence angle varies from 0° to 180°, consistently reaching its maximum value at an incidence angle of 180°. Furthermore, the volume reflectivity increases markedly with higher rainfall rates, whereas its rate of increase attenuates with rising frequency.

The volume reflectivity of raindrops is governed by their backscattering cross-section, which represents the sum of the effective surface areas visible to an observer aligned with the incidence direction of the millimeter-wave (MMW) signal. This interpretation explains why the backscattering cross-section attains relatively high values at both 0° and 180° incidence, corresponding to two extreme geometric conditions. However, at 180° incidence, the contributing raindrop surface is the upper hemisphere, which generally maintains a more spherical and pronounced curvature, resulting in a larger effective scattering area. In contrast, at 0° incidence, the contributing lower hemisphere often exhibits a more oblate spheroidal shape due to aerodynamic flattening, leading to a comparatively reduced effective area. Consequently, the backscattering cross-section reaches its maximum at 180°, a finding that is consistent with numerical simulation results.

Under a fixed operating frequency of 60 GHz, the maximum volume reflectivity values corresponding to rainfall rates of 100 mm/h, 50 mm/h, and 25 mm/h are 4199.10 mm^2^·m^−3^, 2559.73 mm^2^·m^−3^, and 1540.37 mm^2^·m^−3^, respectively. Additionally, under a constant rainfall rate of 100 mm/h, the maximum volume reflectivity values at operating frequencies of 60 GHz, 35 GHz, and 24 GHz are 4199.10 mm^2^·m^−3^, 4015.21 mm^2^·m^−3^, and 2603.92 mm^2^·m^−3^, respectively.

### 3.3. Effects of Incidence Angle Variations in Non-Spherical Raindrops on SCNR

**Figure 8:**
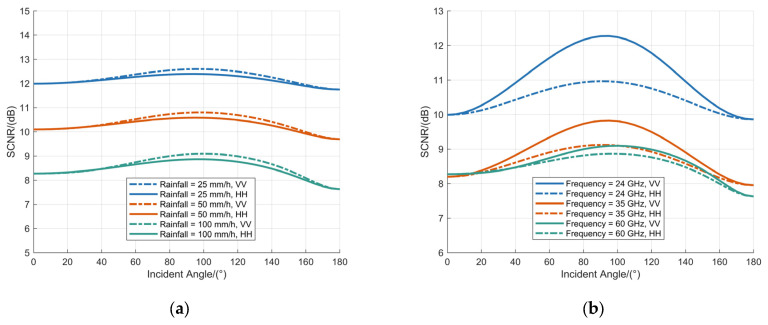
Analysis of SCNR at various raindrop incidence angles: (**a**) the operating frequency is fixed at 60 GHz; (**b**) the rainfall is fixed at 100 mm/h.

The dependence of the incidence angle on the SCNR of MMW signals was examined under two distinct configurations. First, with the fuze operating frequency fixed at 60 GHz, the variation of the SCNR with incidence angle was analyzed for rainfall rates of 100 mm/h, 50 mm/h, and 25 mm/h; the results are presented in Figure 8a. Subsequently, with the rainfall rate fixed at 100 mm/h, the attenuation coefficient was simulated for fuze operating frequencies of 60 GHz, 35 GHz, and 24 GHz, as shown in Figure 8b.

Figure 8 depicts the dependence of the SCNR on the incidence angle for MMW fuze signals interacting with BC model. As the incidence angle increases from 0° to 180°, the SCNR exhibits a non-monotonic trend, characterized by an initial increase to a maximum value followed by a subsequent decrease, reaching its minimum at an incidence angle of 180°. The SCNR demonstrates a pronounced reduction with increasing rainfall rate; however, the magnitude of this reduction attenuates at higher operating frequencies. With the operating frequency fixed at 60 GHz, the minimum SCNR values observed for rainfall rates of 100 mm/h, 50 mm/h, and 25 mm/h are 7.63 dB, 9.69 dB, and 11.75 dB, respectively. Under a constant rainfall rate of 100 mm/h, the corresponding minimum SCNR values for operating frequencies of 60 GHz, 35 GHz, and 24 GHz are 7.63 dB, 7.95 dB, and 8.96 dB, respectively.

### 3.4. Quantitative Analysis of Attenuation Coefficient, Volume Reflectivity, and SCNR of BC Model

A quantitative analysis was conducted to evaluate the influence of incidence angle variation of BC model on the MMW signal attenuation coefficient, volume reflectivity, and SCNR under specified conditions of a 60 GHz operating frequency and a rainfall rate of 100 mm/h. The analysis involved determining the extreme values (maximum and minimum) of these parameters across the full angular range and comparing the differences between them, as summarized in Table 2.

Analysis of the data presented in Table 1 indicates that for the BC model, the attenuation coefficient exhibits symmetry across the incidence angle range from 0° to 180°. Consequently, its maximum and minimum values occur at 180° and 90°, respectively, with a relative difference of 119.28%. In contrast, the volume reflectivity does not demonstrate symmetry, a characteristic attributed to the irregular geometry of the BC model. Its maximum and minimum values are located at 180° and 101°, respectively, differing by 42.27%. The SCNR, as a parameter influenced by both the attenuation coefficient and the volume reflectivity, attains its maximum at 180° and minimum at 101°, exhibiting a variation of 40.28%. The alignment of SCNR extremal values with those of volume reflectivity indicates that the SCNR is more significantly governed by volume reflectivity dynamics.

A comprehensive analysis of multiple operating frequencies and multiple rainfall conditions indicates that both the attenuation coefficient and volume reflectivity reach their maximum values at an incidence angle of 180°, whereas the SCNR attains its minimum value under the same angular condition. This result demonstrates that MMW fuzes experience the most significant signal degradation due to combined attenuation and rain-induced clutter interference at an incidence angle of 180°, establishing this scenario as the most challenging condition for fuze detection performance. To ensure reliable operation throughout the entire detection process—including accurate target initiation and precise ranging—the fuze system must maintain functionality across the full range of possible incidence angles. Consequently, particular emphasis should be placed on investigating the variations in the attenuation coefficient, volume reflectivity, and SCNR for raindrops represented by the BC model, specifically at the critical incidence angle of 180°.

### 3.5. Effects of Drop Size Distribution on Attenuation and Scattering Characteristics

**Figure 9:**
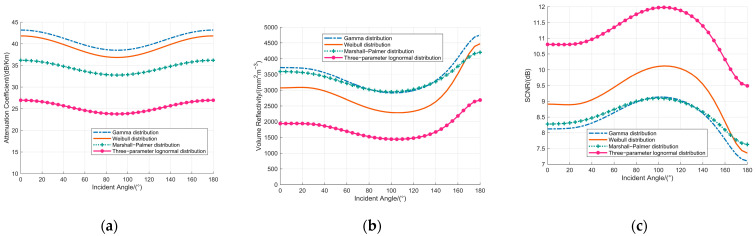
The Effects Analysis of drop size distribution on: (**a**) attenuation coefficient; (**b**) volume reflectivity; (**c**) SCNR.

The DSD quantifies the particle concentration per unit volume across different diameters and is a critical determinant of the attenuation coefficient and volume reflectivity. This study systematically investigates the attenuation coefficient, volume reflectivity, and SCNR under a 60 GHz fuze frequency and a rainfall rate of 100 mm/h, employing multiple DSD models—including the Marshall-Palmer, Weibull [34], gamma [35], and three-parameter lognormal [36] distributions. To ensure an unambiguous comparative analysis among the DSDs, the study is confined to vertically polarized incident waves. The corresponding results are illustrated in Figure 9.

Figure 9a demonstrates that the attenuation coefficient varies significantly with the specific DSD employed. When ranked in descending order of magnitude, the attenuation coefficients correspond to the gamma, Weibull, Marshall-Palmer, and three-parameter lognormal distributions, respectively. Notably, all distributions exhibit a consistent symmetric dependence on the incidence angle: the attenuation coefficient decreases from 0°, attains a minimum at 90°, and subsequently increases toward 180°.

As depicted in Figure 9b, the volume reflectivity under identical incidence angles displays an interleaved pattern across the different DSDs. This behavior originates from substantial disparities in the number concentration of particles across size classes within each distribution, coupled with the variation of the backscattering cross-section with both particle size and incidence angle. Specifically, the volume reflectivity curves for the gamma, Weibull, and Marshall-Palmer distributions intersect within the incidence angle range, while the three-parameter lognormal distribution consistently yields the lowest values. In all cases, the volume reflectivity follows a non-monotonic trend, characterized by an initial decrease followed by an increase with rising incidence angle, and peaks at 180°. At the angle of 180°, the volume reflectivity values, in descending order, correspond to the gamma, Weibull, Marshall-Palmer, and three-parameter lognormal distributions.

According to Figure 9c, the SCNR under the same incidence angle also manifests an interleaved distribution, which is predominantly dictated by the behavior of the volume reflectivity. For each DSD, the SCNR initially increases and subsequently decreases with the incidence angle, reaching its minimum value at 180°. At this critical angle, the SCNR values, ranked from highest to lowest, correspond to the three-parameter lognormal, Marshall-Palmer, Weibull, and gamma distributions.

In conclusion, alterations in the DSD do not modify the fundamental trends of the attenuation coefficient, volume reflectivity, and SCNR as functions of the incidence angle. Consequently, the principal finding—that the attenuation coefficient and volume reflectivity of the millimeter-wave fuze attain their maxima, while the SCNR reaches its minimum at an incidence angle of 180°—is robust and applicable across the various DSDs investigated.

## 4. MMW Fuze Echo Characteristics in Rainfall Environments Based on the BC Model at an Incidence Angle of 180°

Based on the worst-case analysis method, the incidence angle of 180° was identified as the most severe incidence angle for the BC model. Consequently, the attenuation coefficient, volume reflectivity, and SCNR of the BC model were therefore analyzed at this angle. A comparative analysis was subsequently conducted with the SP model and the AE model, both also evaluated at an incidence angle of 180°.

Analysis of Figure 4 and Figure 6 indicates that the attenuation and backscattering characteristics of the SP model are independent of the incidence angle. In contrast, the AE model exhibits symmetry in its attenuation and backscattering properties across the incidence angle range from 0° to 180°. The attenuation coefficient and volume reflectivity attain their minimum values at an incidence angle of 90°, while reaching their maximum values at 0° and 180°. Therefore, at an incidence angle of 180°, the attenuation coefficient and volume reflectivity of the BC, AE, and SP models all reach their maximum values, enabling a direct and equitable comparison among them.

### 4.1. Analysis of Attenuation Coefficient Based on the BC Model at an Incidence Angle of 180°

**Figure 10:**
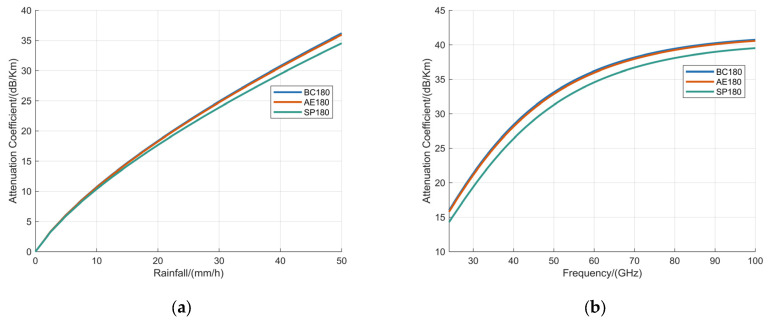
Analysis of attenuation coefficient based on BC180 model, AE180 model, and SP180 model with: (**a**) the operating frequency fixed at 60 GHz; (**b**) the rainfall fixed at 100 mm/h.

The dependence of the attenuation coefficient on the rainfall rate was examined for the BC, AE, and SP raindrop models at an incidence angle of 180°, with the operating frequency fixed at 60 GHz; the corresponding results are illustrated in Figure 10a. Subsequently, with the rainfall rate maintained constant at 100 mm/h, the variation of the attenuation coefficient as a function of operating frequency was evaluated for the same models and incidence angle, as depicted in Figure 10b. The abbreviations BC180, AE180, and SP180 denote the BC model, AE model, and SP model evaluated at an incidence angle of 180°.

Figure 10 demonstrates that the attenuation coefficients of the three raindrop models exhibit a rapid increase with rising rainfall intensity and operating frequency of the MMW fuzes. The BC180 model and AE180 model show minimal differences in their attenuation characteristics, indicating a high degree of similarity in their response. In contrast, both models differ significantly from the SP180 model, suggesting that the simplified spherical model inadequately represents the actual attenuation properties of non-spherical raindrop particles.

### 4.2. Analysis of Volume Reflectivity Based on the BC Model at an Incidence Angle of 180°

**Figure 11:**
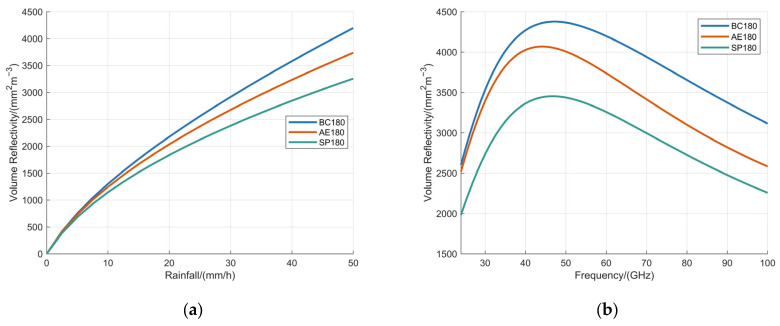
Analysis of volume reflectivity based on BC180 model, AE180 model, and SP180 model with: (**a**) the operating frequency fixed at 60 GHz; (**b**) the rainfall fixed at 100 mm/h.

The dependence of volume reflectivity on rainfall rate was analyzed for the BC180 model, AE180 model, and SP180 model under the condition of a fixed operating frequency of 60 GHz, with the results detailed in Figure 11a. Following this, an assessment of how volume reflectivity varies with operating frequency was conducted for these models under a constant rainfall rate of 100 mm/h, as illustrated in Figure 11b.

Figure 11 depicts that the volume reflectivity for all three models exhibits a rapid increase with rising rainfall intensity. In contrast, under constant rainfall conditions, the volume reflectivity initially rises to a maximum and subsequently undergoes a gradual decline as the operating frequency increases. This trend indicates that elevating the fuzes operating frequency can effectively mitigate rain-induced clutter under fixed rainfall intensity. Among the three models, the BC180 model consistently exhibited the highest volume reflectivity values, whereas the SP180 model demonstrated the lowest. Considerable discrepancies are observed among the three models, demonstrating that both the AE model and the SP model inadequately represent the volume reflectivity characteristics of realistic BC model.

### 4.3. Analysis of SCNR Based on the BC Model at an Incidence Angle of 180°

**Figure 12:**
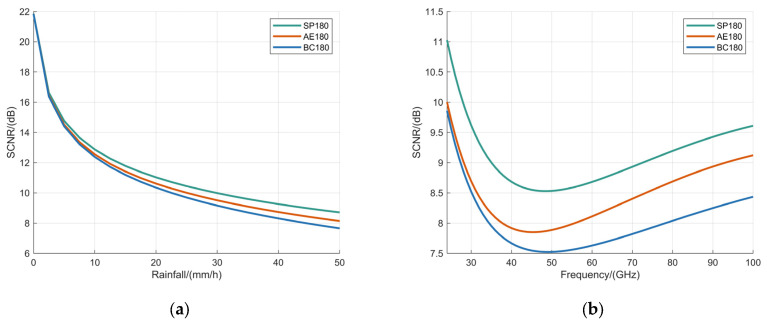
Analysis of SCNR based on BC180 model, AE180 model, and SP180 model with: (**a**) the operating frequency fixed at 60 GHz; (**b**) the rainfall fixed at 100 mm/h.

The dependence of SCNR on rainfall rate was analyzed for the BC180 model, AE180 model, and SP180 model under the condition of a fixed operating frequency of 60 GHz, with the results detailed in Figure 12a. Following this, an assessment of how SCNR varies with operating frequency was conducted for these models under a constant rainfall rate of 100 mm/h, as illustrated in Figure 12b.

Figure 12 demonstrates the variation of the SCNR as a function of rainfall intensity and operating frequency for the three raindrop models. The SCNR exhibits a rapid decrease with increasing rainfall intensity; specifically, as the rainfall rate rises from 0 mm/h to 100 mm/h, the SCNR declines by approximately 14.21 dB. In contrast, with increasing operating frequency, the SCNR initially decreases to a minimum value before gradually increasing. Among the three models, the BC180 model consistently exhibited the lowest SCNR values, whereas the SP180 model demonstrated the highest.

### 4.4. Quantitative Analysis of Attenuation Coefficient, Volume Reflectivity, and SCNR for the BC Model, AE Model, SP Model

To quantitatively analyze the discrepancy of different raindrop models on the attenuation coefficient, volume reflectivity, and SCNR of MMW signals, a specific scenario with an operating frequency of 60 GHz and a rainfall rate of 100 mm/h was examined. The specific values and differences in these parameters for three distinct raindrop models are summarized in Table 3.

Analysis of the data presented in Table 3 reveals significant disparities in key parameters among the SP, AE, and BC raindrop models. Regarding the attenuation coefficient, the discrepancies between the BC model and the SP and AE models are 45.88% and 5.93%, respectively, indicating a close agreement between the AE and BC models. For volume reflectivity, the differences between the BC model and the SP and AE models are 28.27% and 12.31%, respectively. Similarly, for the SCNR, the corresponding differences are 27.35% and 11.69%. These results collectively demonstrate that the simplified AE and SP models exhibit substantial deviations from the more physically representative BC model.

## 5. Conclusions

This study systematically investigates the dynamic incidence angles effects of non-spherical raindrops on rain attenuation and scattering for MMW fuzes. The principal findings and implications are summarized as follows:

First, the permissible range of the incidence angle for interactions between the MMW fuze and raindrops was established based on a synthesis of the fuze’s flight path, its beamwidth, and the distribution of raindrop tilt angles. Utilizing the IIM T-matrix method, the attenuation coefficient and volume reflectivity of the MMW signal were rigorously modeled. Accounting for the spatially non-uniform distribution of rain clutter, a modified SCNR was formulated by integrating clutter contributions from multiple range cells preceding the target detection cell.

Second, simulation results demonstrate a strong dependence of the attenuation coefficient, volume reflectivity, and SCNR on the incidence angle for BC model, with maximum relative disparities of 119.28%, 42.27%, and 40.28% observed across the 0° to 180° spectrum, respectively. Of critical importance, the 180° incidence angle was identified as the most challenging scenario, where attenuation and reflectivity peak concurrently with an SCNR minimum, thereby severely compromising fuze detection performance. The validity of this conclusion is maintained irrespective of the specific drop size distribution employed.

Finally, under this worst-case angle (180°), the attenuation coefficient and volume reflectivity show a positive correlation with increasing rainfall rate. Regarding frequency dependence, attenuation increases monotonically with frequency, while volume reflectivity and SCNR demonstrate non-monotonic trends. A key finding is that compared to the SP model, the BC model yields substantial deviations at 180° incidence: +45.88% in attenuation, +28.27% in volume reflectivity, and −27.35% in SCNR. These significant discrepancies underscore the critical importance of employing realistic non-spherical models over simplified spherical approximations for accurate performance prediction.

In summary, this work establishes a theoretical foundation for calibrating design margins and optimizing anti-interference strategies in MMW fuzes operating in complex rainfall environments. The proposed integrated framework, combining a realistic raindrop model with a worst-case analysis under dynamic incidence angles, provides a more reliable assessment tool that effectively addresses the limitations of conventional static analyses.

## Figures and Tables

**Table 1:** Simulation parameter values.

Parameter	Value	Parameter	Value
Operating frequency f	24–100 GHz	Transmit power $P_{t}$	12 dBm
Fuze bandwidth B	500 MHz	Antenna gain G	3 dB
Incidence Angle θ	0–180°	RCS of target $\sigma_{f}$	10 dBsm
3 dB beamwidth α	120°	Target distance R	12 m
Rainfall I	0–100 mm/h	Boltzmann constant $k_{0}$	$1.38\times 10^{-23}\;J/K$
Equivalent diameter of raindrop D	0–8 mm	System loss $L_{s}$	7 dB
Raindrop diameter interval dD	0.2 mm	Equivalent operating bandwidth $B_{n}$	50 KHz
Temperature T	20 °C	Noise Figure $F_{n}$	3.5 dB

**Table 2:** Value of attenuation coefficient, volume reflectivity, and SCNR of BC model at 60 GHz operating frequency and 100 mm/h rainfall rate.

Parameter	Extreme Value	Incidence Angle	Value	Actual Fluctuation Value	Relative Fluctuation Value
attenuation coefficient	Min value	90°	32.78 dB/Km	3.41 dB/km	119.28%
	Max value	180°	36.19 dB/Km		
volume reflectivity	Min value	101°	2947.41 mm^2^·m^−3^	1251.69 mm^2^·m^−3^	42.27%
	Max value	180°	4199.10 mm^2^·m^−3^		
SCNR	Min value	180°	7.63 dB	1.47 dB	40.28%
	Max value	101°	9.10 dB		

**Table 3:** Value of attenuation coefficient, volume reflectivity, and SCNR for the BC180 model, AE180 model, SP180 Model.

Parameter	Model	Value	Actual Difference from BC180 Model	Relative Difference from BC180 Model
attenuation coefficient	SP180	34.55 dB/Km	−1.64 dB/Km	−45.88%
	AE180	35.94 dB/Km	−0.25 dB/Km	−5.93%
	BC180	36.19 dB/Km	/	/
volume reflectivity	SP180	3258.52 mm^2^·m^−3^	−940.58 mm^2^·m^−3^	−28.87%
	AE180	3738.96 mm^2^·m^−3^	−460.14 mm^2^·m^−3^	−12.31%
	BC180	4199.10 mm^2^·m^−3^	/	/
SCNR	SP180	8.68 dB	+1.05 dB	+27.35%
	AE180	8.11 dB	+0.48 dB	+11.69%
	BC180	7.63 dB	/	/

## Data Availability

The data are available from the corresponding author upon reasonable request.

## Abbreviations

The following abbreviations are used in this manuscript:

MMW	Millimeter-wave
IIM	Invariant imbedding
SCNR	Signal-to-clutter-plus-noise ratio
BC model	Beard and Chuang model
DDA	Discrete dipole approximation
AE model	Approximate ellipsoidal model
SP model	Spherical model
RCS	Radar cross-section
CFAR	Constant False Alarm Rate
DSD	Drop size distribution
BC180	Beard and Chuang model at an incidence angle of 180°
AE180	Approximate ellipsoidal model at an incidence angle of 180°
SP180	Spherical model at an incidence angle of 180°
